# A Longitudinal Study of Individual Radiation Responses in Pediatric Patients Treated with Proton and Photon Radiotherapy, and Interventional Cardiology: Rationale and Research Protocol of the HARMONIC Project

**DOI:** 10.3390/ijms24098416

**Published:** 2023-05-08

**Authors:** Maria Grazia Andreassi, Nadia Haddy, Mats Harms-Ringdahl, Jonica Campolo, Andrea Borghini, François Chevalier, Jochen M. Schwenk, Brice Fresneau, Stephanie Bolle, Manuel Fuentes, Siamak Haghdoost

**Affiliations:** 1 CNR National Research Council Institute of Clinical Physiology, 56125 Pisa, Italy; andreassi@ifc.cnr.it (M.G.A.); aborghini@ifc.cnr.it (A.B.); 2 Radiation Epidemiology Team, Center for Research in Epidemiology and Population Health, INSERM U1018, Gustave Roussy, Université Paris-Saclay, 94805 Villejuif, France; nadia.haddy@gustaveroussy.fr; 3 Department of Molecular Biosciences, The Wenner-Gren Institute, Stockholm University, 10691 Stockholm, Sweden; mats.harms-ringdahl@su.se; 4 CNR National Research Council Institute of Clinical Physiology, ASST Grande Ospedale Metropolitano Niguarda, 20162 Milan, Italy; jonica.campolo@cnr.it; 5 UMR6252 CIMAP, CEA-CNRS-ENSICAEN-University of Caen Normandy, 14000 Caen, France; francois.chevalier@cea.fr; 6 Advanced Resource Center for HADrontherapy in Europe (ARCHADE), 14000 Caen, France; 7 Affinity Proteomics, SciLifeLab, School of Engineering Sciences in Chemistry, Biotechnology and Health, KTH—Royal Institute of Technology, 10044 Stockholm, Sweden; jochen.schwenk@scilifelab.se; 8 Department of Children and Adolescents Oncology, Gustave Roussy, Université Paris-Saclay, 94805 Villejuif, France; brice.fresneau@gustaveroussy.fr; 9 Cancer and Radiation Team, Center for Research in Epidemiology and Population Health, INSERM U1018, Gustave Roussy, Université Paris-Saclay, 94805 Villejuif, France; 10 Department of Radiation Therapy, Gustave Roussy, Université Paris-Saclay, 94805 Villejuif, France; 11 Deparment of Medicine and General Service of Cytometry, Proteomics Unit, Cancer Research Centre-IBMCC, CSIC-USAL, IBSAL, Campus Miguel de Unamuno s/n, University of Salamanca-CSIC, 37007 Salamanca, Spain

**Keywords:** radiotherapy, ionizing radiation, proton radiotherapy, pediatric oncology, HARMONIC project, congenital heart disease, cardiac catheterization, radiation biomarkers

## Abstract

The Health Effects of Cardiac Fluoroscopy and Modern Radiotherapy (photon and proton) in Pediatrics (HARMONIC) is a five-year project funded by the European Commission that aimed to improve the understanding of the long-term ionizing radiation (IR) risks for pediatric patients. In this paper, we provide a detailed overview of the rationale, design, and methods for the biological aspect of the project with objectives to provide a mechanistic understanding of the molecular pathways involved in the IR response and to identify potential predictive biomarkers of individual response involved in long-term health risks. Biological samples will be collected at three time points: before the first exposure, at the end of the exposure, and one year after the exposure. The average whole-body dose, the dose to the target organ, and the dose to some important out-of-field organs will be estimated. State-of-the-art analytical methods will be used to assess the levels of a set of known biomarkers and also explore high-resolution approaches of proteomics and miRNA transcriptomes to provide an integrated assessment. By using bioinformatics and systems biology, biological pathways and novel pathways involved in the response to IR exposure will be deciphered.

## 1. Introduction

The use of ionizing radiation (IR) in medicine represents significant benefits for the medical care of patients. Indeed, IR remains one of the major therapeutic options for cancer treatment [1]. IR is also used for diagnostic and therapeutic imaging, particularly for pediatric patients with congenital or acquired heart disease, who may receive one or more cardiac catheterization procedures as part of their management [2,3,4]. While benefits for the patients largely outweigh the risk, the potential adverse health effects of exposure to IR are particularly important to be explored in populations of young patients who are more radiosensitive and, nowadays, survive their disease for decades [3,4]. 

IR is a well-known risk factor for cancer induction, and recent studies support the existence of an excess cancer risk, even at low doses of radiation [5]. Children are especially vulnerable to the oncogenic effects of IR. Moreover, the oncogenic effects of IR require a long latent period (from years to decades) that varies with the type of malignancy; therefore, an infant or child has a longer lifetime risk for developing radiation-induced cancers than an adult. Radiation-induced second malignancies are one of the most serious adverse effects following radiotherapy of primary cancers in childhood cancer [6]. Typically, radiation-induced malignancies develop in normal tissue within radiotherapy fields with a latency period of 5–10 years for hematologic malignancies and 10–60 years for solid tumors [6]. Many clinical studies reported an increased risk for second primary cancer, histopathologically different from the first tumor, in organs inside as well as outside the primary beam [6]. Interestingly, it was reported that leukemias and carcinomas are more often seen in organs receiving low-dose radiation (out-of-field dose), whereas sarcomas are more common in tissues or organ receiving high-dose radiation (in-field doses) [7]. However, the exact mechanism and dose–response relationship for radiation-induced malignancy, for both in-field and out-of-field doses, are not well understood; thus, it is necessary to investigate how radiotherapy, photons, and protons impact carcinogenic risk in childhood cancer management [7]. For instance, the therapeutic use of proton beams has the potential to provide a better depth–dose profile and remarkable reduction in the dose to the adjacent normal tissues compared with photon beams [7]. Additionally, there is growing evidence supporting an increased risk for late adverse non-cancer conditions [8], including cardiac and vascular effects. Thus, late adverse effects of radiotherapy have been observed on large vessels, causing cerebrovascular [9,10] and cardiovascular [11,12] diseases. 

Nowadays, there is also evidence for a significant elevation of cancer risk in patients with acquired as well as congenital heart diseases (CHD) in response to repeated radiological exposures [13,14]. However, more data are needed to better define the “malignant price of cardiac care” [15].

The risk estimates of long-term health effects of low doses of IR (cancer and non-cancer) are still incomplete, particularly for pediatric patients. Large patient cohorts, extended follow-up, validated clinical data, and reliable dosimetry for the cohorts are needed to address this challenge. The integration of epidemiological and biological research through panels of biomarkers, together with a mechanistic understanding of the cellular responses to a particular dose and radiation quality, will provide powerful means to improve risk estimates, leading to a better quantification of the magnitude of risks associated with low-dose exposures, e.g., for out-of-field organs.

The Health Effects of Cardiac Fluoroscopy and Modern Radiotherapy in Pediatrics (HARMONIC) is a five-year project funded by the European Commission to improve understanding of the long-term health risks from medical ionizing radiation exposure in children and young patients (https://harmonicproject.eu/, accessed on 4 March 2023). The HARMONIC project uses an integrated approach of conventional epidemiology complemented by non-invasive imaging and molecular epidemiology to assess cancer and non-cancer outcomes in pediatric patients treated with modern radiotherapy techniques (such as proton therapy) for cancer and X-ray-guided interventional catheterization procedures for CHD.

The purpose of the manuscript is to describe the bioanalytical research goals of the HARMONIC project, the rationale, and the study design, including the enrolment, endpoints, and expected results of the study.

The general objective of the bioanalytical studies of the HARMONIC project is to provide a mechanistic understanding of the molecular pathways and the cellular responses that are triggered by the medical applications of IR in pediatric patients.

A mechanistic understanding, together with the identification of biomarkers for individuals at increased risk to develop adverse health effects, has the potential to increase the power of epidemiological studies regarding health effects caused by IR [16,17,18]. Such biomarkers may be useful in identifying susceptible individuals who are more vulnerable to radiation damage for whom individualized treatment can be considered (by radiation sparing policy or attempts to pharmacologic or dietary radioprotection).

The specific aims are to:

- identify radiation-induced biochemical responses in blood and saliva from pediatric patients exposed to medical IR;
- evaluate dose–response relationships for different radiation qualities and delivery techniques with regards to specific biochemical responses;
- search for pre-existing biomarkers of radiation sensitivity and health effects that may be useful for molecular epidemiological studies to identify patients with a potential higher risk of radiation-induced adverse health effects.

## 2. Experimental Design 

The HARMONIC biological study is a prospective observational study which aims to investigate the biological changes induced by ionizing radiation exposure at various time points before and after exposure. It will focus on specific molecular biomarkers reported as ‘early signs’ of biological damage and long-term health effects. These include biomarkers of oxidative stress (8-hydroxy-2′-deoxyguanosine) [19,20,21], protein markers of inflammation (PTX3, IL-6, IL-10, TNF-α, NF-kB, MCP-1, etc.) [22,23], and genetic markers (telomere shortening and mtDNA copy numbers) [24,25,26,27,28,29]. 

To decipher significant intracellular pathways and novel potential biomarkers involved in response to the radiation regimes applied, four different approaches will be used: multiplexed protein profiling assays on blood plasma [30], reverse-phase protein array (RPPA) on proteins isolated from peripheral blood mononuclear cells [31], miRNA transcriptome on whole blood and saliva [32], and liquid chromatography–mass spectrometry (LC–MS) on saliva.

**Figure 1:**
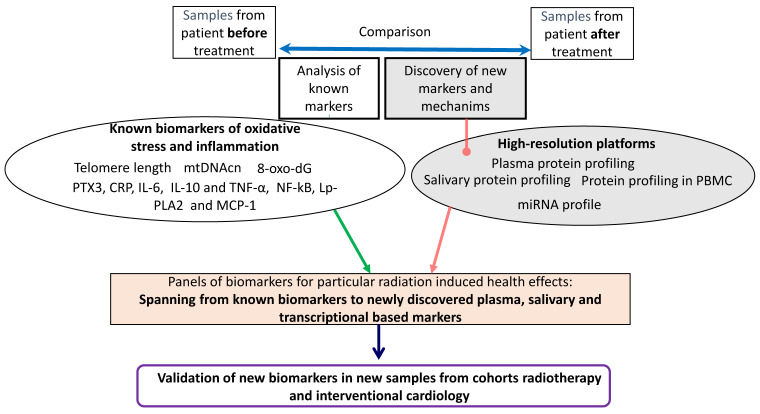
Overall strategy of the planned biological research in the HARMONIC project.

Finally, we will develop and implement new bioinformatic models to integrate the collected biological, clinical, and dosimetry data. that may be used in epidemiological and clinical approaches to identify patients at higher risk for radiation-induced adverse health effects, not only before starting (by analyzing the sample taken before exposure), but also after finishing the exposure (by analyzing samples taken after exposure). Biomarkers will be studied in both blood and saliva to investigate whether saliva can be used as a non-invasive sample to analyze biomarkers in large-scale molecular epidemiology studies. The overall strategy of the “biology” project is presented in Figure 1.

## 3. Material and Equipment

### 3.1. Study Population

This exploratory study will include 150 patients: 50 patients treated for cancer with proton therapy, 50 patients with photon therapy, and 50 patients treated with X-ray-guided interventional catheterization procedures for CHD. Specific inclusion and exclusion criteria are listed in Table 1.

Ethics approval has been already obtained in all participating centers. All eligible patients received an information brochure and are invited to participate in the study by the responsible physician. Informed consent is signed by the patient or his/her legal representative before entering the study. Detailed demographic, clinical and treatment data are retrieved by the attending physician and from the patient’s medical electronic record. Data protection officers (DPOs) from each organization will be involved to ensure compliance with General Data Protection Regulations (GDPRs).

### 3.2. Biological Sample Collection

**Figure 2:**
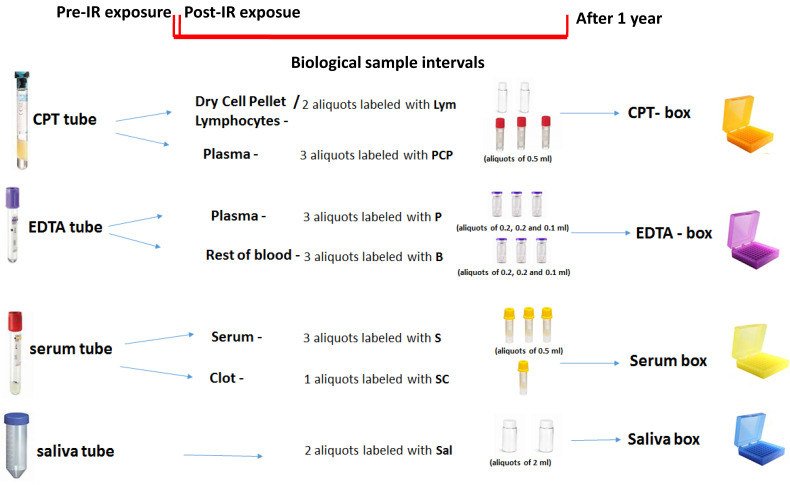
Study design of the Harmonic project and overview of biological sample collection.

For radiotherapy patients, blood and saliva will be collected at three time points: before radiotherapy; three months after the last fraction (time point for the first follow-up); and one year after completion of the treatment. For X-ray-guided interventional catheterization procedures, biological samples will also be collected at three time points: before intervention; the same day after completion; and one year after completion. Figure 2 summarizes the protocol for the collection, preparation, and storage of biosamples.

Briefly, a maximum of 12 mL blood will be collected at each time point in three different tubes, which are as follows:

- one BD vacutainer^®^ CPT™ tube for the isolation of lymphocytes (~4 mL);
- one vacutainer tube containing EDTA K2 (~4 mL);
- one clot activator serum separation tube (~4 mL).

The samples will be given a unique patient identification number (pseudonymization). Within two hours post collection, tubes with blood samples will be centrifuged according to standard operating procedures (SOPs) prepared by the HARMONIC consortium to obtain lymphocytes, serum, and plasma. To investigate the possible impact of pre-analytical variables, we will record and share information on the study centers, time, and calendar days when the samples are collected. We will also record and share the time that elapses between the blood draw, centrifugation, and first freezing. 

Regarding saliva samples, approximately 4–5 mL of saliva will be collected at each time point in a sterile 10 mL plastic tube without any additive. Saliva samples will be divided into two aliquots, 2 mL in each. Aliquots of biological samples from each donor will be immediately stored at −80 °C (Figure 2). The type of tubes, volume, and number of each aliquots are summarized in Figure 2. At specific time points in the project, coded samples will be shipped from a respective clinic on dry ice to a centralized Biobank, where they will be stored at −80 °C until use. 

### 3.3. Biological Measures

The samples will be analyzed by state-of-the-art methods to determine the levels of selected biomarkers. Moreover, the samples will be examined by innovative high-throughput approaches, including analyses of miRNA transcriptomes and proteomics [30,31,32]. The handling and analytical procedures will follow the respective SOP procedures for all biologic sampling, handling, shipping, and analysis.

## 4. Detailed Procedure

### 4.1. 8-Hydroxy-2′-deoxyguanosine (8-oxo-dG) and Markers of Inflammation

The levels of 8-oxo-dG in serum and saliva will be determined using an ELISA method where the samples are essentially purified by Bond Elute columns, as previously described [19,20]. Briefly, 800 µL blood serum or saliva will be purified using a C18 solid phase Bond Elut extraction column. The purified samples will be freeze-dried and reconstituted in PBS. Then, 300 µL of the purified sample will be mixed with 150 µL of primary antibody against 8-oxo-dG and distributed in three wells of a 96-well ELISA plate pre-coated with 8-oxo-dG and then incubated at 37 °C for 120 min. Secondary antibody body will then be added followed by the staining solution in order to quantify the yield of secondary antibodies bounded to primary antibodies in each well using a 96-well automatic ELISA plate reader. Each sample will be analyzed in triplicate. A standard curve for 8-oxo-dG (0.05–10 ng/mL) will be established for each plate and the concentration of 8-oxo-dG in each sample will be calculated based on the standard curve.

### 4.2. Analysis of Telomere Length (TL) and mtDNA Copy Number (mtDNA-CN)

TL and mtDNA-CN will be measured on DNA extracted from 200 µL of biological samples of blood and saliva samples by real-time PCR (CFX384 Touch™ Real-Time PCR System, Bio-Rad Life Sciences) according to standardized protocols [25,26]. Briefly, TL will be measured in genomic DNA by determining the ratio of a telomere repeat copy number (T) to a single-copy gene (S) and copy number (T/S ratio). The relative telomere length will be calculated using the following formula “T/S ratio = 2−ΔΔCt”, where ΔCt = Ct telomere − a Ct single-copy gene. The T/S ratio reflects the average length of the telomeres across all leukocytes. For the quantification of mtDNA-CN, the NDI1 gene in the undeleted region for the reference sequence of mtDNA will be used as an internal control (mtNDI1) and human ß-globin gene of genomic DNA (gDNA) will be amplified by PCR in both gDNA and mtDNA. ΔCt values will be calculated from the difference between the Ct for the ß-globin gene and the Ct for the NDI1 gene and used to measure mtDNA-CN relative to gDNA. mtDNA-CN will be calculated using the (2ΔCt) method (ΔCt = Ct mtNDI1 − CtgDNA). 

### 4.3. miRNA Profiling Analysis

Total RNA will be isolated from 500 µL of blood and saliva samples using a RiboPure™-Blood Kit (ThermoFisher, Waltham, MA, USA) and a miRNeasy Serum/Plasma Kit (QIAGEN, Hilden, Germany), respectively, according to the manufacturer’s protocol [32]. The expression profiling of miRNAs will be analyzed using the Illumina MiSeq platform. For each patient, we will carry out a small RNA sequencing experiment to characterize the different miRNA expression profiles in samples for each time point. Prepared libraries will be run on Miseq, and miRNA identification and dysregulated expression analyses will be performed using latest version of iMir software (https://www.labmedmolge.unisa.it/italiano/home/imir, accessed on 5 September 2022), a fully automated workflow for the rapid analysis of high-throughput small RNA-Seq data. Specific dysregulated miRNAs will be further validated using qRT-PCR with sequence-specific TaqMan microRNA assays and a TaqMan Universal PCR Master Mix, as opposed to AmpErase UNG (Thermo Fisher Scientific, USA), in accordance with the manufacturer’s instructions. The miRNA expression levels will be normalized to the U6 small nuclear RNA and calculated using the ΔΔCt method [32]. The target genes from differentially expressed miRNAs will be predicted using DIANA miRPath software (microrna.gr/mirpath, accessed on 5 September 2022). Then, the targets will then be further analyzed for gene ontology (GO) function enrichment terms (geneontology.org/, accessed on 5 September 2022), Kyoto Encyclopedia of Genes Genomes (KEGG) pathway classification (www.genome.jp/kegg/, accessed on 5 September 2022), and Reactome pathway databases (www.reactome.org, accessed on 5 September 2022).

### 4.4. Plasma Protein Profiling

Briefly, from the literature, a list of 90 protein markers that have previously been identified as potential markers of diseases related to the late effects of radiation exposure, particularly vascular diseases and secondary cancer, will be established. Aliquots of 100 µL plasma will be used for plasma proteome analysis using Olink’s affinity proteomics platform. The approach is based on paired antibodies, coupled to unique and partially complementary oligonucleotides, and measured by quantitative real-time PCR. This dual-recognition DNA-coupled method provides high specificity and sensitivity for an analysis of at least 90 proteins in parallel [30]. We plan to analyze the 98 selected proteins in all samples from the three cohorts. Different statistical and computational models for single and multivariate analysis will then be used to identify the modified pathways.

### 4.5. Reverse-Phase Protein Arrays (RPPAs)

Reverse-phase protein arrays (RPPAs) will allow us to study protein expression levels and the activation status of cell signaling pathways. Isolated peripheral blood mononuclear cells (PBMCs) from blood collected into CPT tubes will be analyzed by customized RPPA (Proteomics Unit. IBSAL. University of Salamanca). To summarize, the cells will be lysed and the protein extract will be serially diluted with a protein lysis buffer, supplemented with proteases and phosphatase inhibitors. Five serial dilutions/sample, ranging from 2000 to 125 µg/mL, and two technical replicates per dilution will be applied on the nitrocellulose microarray membrane. In addition, a few spike-in proteins as RPPAs, such as negative and positive controls, will be included. 

The membranes will be incubated with primary antibodies that target proteins of interest or without primary antibodies as negative controls. All primary antibodies for RPPA screening have been previously tested by Western blotting to assess their specificity and selectivity to the targeted protein. RPPA readout is a fluorescent signal correlated to the protein expression level. Samples will be applied on membranes in three technical replicates (spots), and the membranes will then be individually incubated with antibodies targeting one protein of interest, followed by an incubation with a fluorescent dye. Fluorescent signals will be acquired by a microarray scanner at high resolutions and minimal auto-fluorescent background. The NormaCurve method will be used for data quantification and normalization [31]. This method includes a normalization for (i) background fluorescence, (ii) variations in the total amount of spotted protein; and (iii) spatial bias on the membranes. The normalized values will be employed to compare the protein expression levels across samples. Briefly, for each spot, the raw fluorescent signal of the proteins will be corrected with the fluorescent signal of the negative control (signal obtained after incubating an array, without the targeted protein antibody). This corrected signal will be divided by the total amount of spotted protein, corresponding to the normalized signal. Finally, the normalized signals of all the proteins will be scaled according to the median for further comparisons and statistical analysis. For each sample, one value will be generated for each targeted protein, and further statistical analysis will be considered. 

### 4.6. Saliva Protein Analysis 

The saliva protein concentrations will be determined using a colorimetric protein assay (BCA Protein Assay Kit, Thermo Scientific, Waltham, MA, USA). The proteomic workflow is as follows. The iST-BCT Kit (PreOmics, Martinsried, Germany) will be used to perform a fast, reliable, and reproducible sample preparation on all the patient samples. Fifty microliters of saliva will be used as the starting material (the volume will be adjusted depending on the BCA result). Saliva proteins will be precipitated with 200 μL of ethanol at −20 °C overnight. Samples will then be centrifuged (at 17,000× *g* for 5 min at 4 °C) and the supernatants will be removed. Salivary protein pellets will be re-suspended, lysed, reduced, and alkylated in 10 min at 95 °C. Proteins will be digested in one hour. Generated peptides will be cleaned before LC-MS injection. Purified tryptic digests will be separated with a predefined 60 SPD method (21 min gradient time and 200 ng peptides) on an Evosep One LC system (Evosep, Odense, Demmark). A fused silica 10 μm ID emitter (Bruker Daltonics, Waltham, MA, USA) is placed inside a nanoelectrospray source (CaptiveSpray source, Bruker Daltonics, Waltham, MA, USA). The emitter is connected to a 8 cm × 150 μm reverse-phase column, packed with 1.5 μm C18 beads. Mobile phases will comprise water and acetonitrile, buffered with 0.1% formic acid. The column will be heated to 40 °C in an oven compartment. LC is coupled online to a TIMS Q-TOF instrument (timsTOF Pro 2, Bruker Daltonics) with a diaPASEF acquisition method. Samples will be acquired using a diaPASEF method, consisting of 12 cycles, including a total of 34 mass width windows (25 Da width, from 350 to 1200 Da) with 2 mobility windows each, leaving a total of 68 windows that cover the ion mobility range (1/K0) from 0.64 to 1.37 V s/cm^2^. Saliva proteins will be quantified using a label-free DIA approach with DIA-NN software (https://github.com/vdemichev/diann, accessed on 4 March 2023). DIA-NN version 1.8 will be used first to build an in silico predicted library from the human FASTA database (NextProt 2022-02-25), enabling the ‘FASTA digest for library-free search/library generation’ and ‘Deep learning-based spectra’ options, as well as RTs and IMs prediction. The predicted library will be used to analyze the diaPASEF dataset.

### 4.7. Radiation Doses Data

For each patient, the average whole-body dose or mean/maximum dose and non-target organ (out-of-field organ) doses will be estimated in collaboration with physicists responsible for dosimetry studies in the Harmonic project (https://harmonicproject.eu, accessed on 4 May 2023). Briefly, the strategy for dose estimation will rely on Monte Carlo simulations for CHD patients and on treatment planning systems and analytical models for cancer patients [33]. These strategies were benchmarked against measurements on physical phantoms and reference Monte Carlo simulations [34,35]. As we are analyzing plasma proteins, the total dose to the blood will also be estimated and considered.

### 4.8. Integrative Analysis of Biological Function and Networks

Integrative data analysis of multiple sets of data types will be performed to construct an interaction network of differentially expressed features (miRNAs and proteins) to elucidate the molecular mechanisms underlying the biological response to IR and to discover new potential biomarkers. In brief, each dataset from the different independent analyses (miRNA transcriptome sequencing and proteomics) will first be analyzed in relation to the available clinical parameters, e.g., age, sex, diagnosis, and background diseases, to identify significantly different features between the baseline and post-IR exposure responses. Then, the radiation-deregulated miRNAs and proteins will be analyzed by an integrative procedure using software, such as ingenuity pathway analysis (Qiagen Bioinformatics; Redwood City, CA, USA; www.qiagen.com/ingenuity, accessed on 5 March 2023), in order to identify the most significantly affected pathways, their components, and associated signaling networks. 

### 4.9. Sample Size and Plan for Statistical Analysis

This study is exploratory as the number of available patients is limited. With the use of data from our previous study on leukocyte telomere length [25], priori power analysis (Spearman’s correlation test) requires a sample size of 34 patients to achieve >80% power (alpha = 0.05) and to detect an effect size of 0.5 (G*Power, version 3.1.9.2). We plan to include a target population of 100 patients from each cohort, considering the feasibility aspects of the study, the estimated level of recruitment in each participating clinical site, and a drop-out rate of 50%, aiming for a minimum of 50 participating patients in each cohort. Concerning the statistical plan, a database including clinical (diagnosis, different therapies, CT and MRI images, etc.), dosimetric, and experimental data for each patient will be created in order to perform appropriate statistical analysis. Descriptive data will be presented as frequencies with proportions for categorical variables, and either as means with corresponding SDs or medians with corresponding IQRs for continuous variables depending on the distribution. Statistical tests will include Pearson’s x^2^ test for frequencies, the Mann–Whitney U test for non-normally distributed continuous variables, and Student’s *t*-test for normally distributed variables. Spearman’s correlation test will be used to explore the association between variables and radiation doses. 

Exploratory analysis, including unsupervised clustering and principal component analysis, will also be performed to stratify patients according to differential protein expression or relative protein abundance. Other soft clustering, dimensionality reduction (e.g., tSNE and UMAP), or unsupervised analyses (e.g., group-based trajectory models) will be conducted to identify groups of individuals that follow similar shifts or trends on protein relative abundance or differential protein expressions over time, considering that all time points will be performed. The differential profiles will be determined based on the data analysis for baseline and each timepoint, as well as changes from baseline values (relative and absolute change). 

A mixed-effects model will be used to study the association between different biomarkers at different time points and the dosimetry data (dose, volume, and beam quality). Lastly, the differences in biomarker levels between a follow-up timepoint and baseline will be studied as a function of dosimetric indicators using general linear models, with adjustment for potential confounders (chemotherapy, disease history, medicines, BMI, etc.). Statistical significance for all analyses will be assessed using two-sided tests with an alpha level of 0.05 and adjustment for multiple comparisons.

## 5. Expected Results

The HARMONIC project addresses a crucial question regarding the health risks for pediatric patients exposed to ionizing radiation from radiotherapy or interventional cardiology (UNSCEAR report 2008) [1,2,3,4]. Over the recent years, there has been considerable technological advancements that improve the therapeutic gain of radiotherapy, i.e., maximizing the dose to the tumor while sparing the healthy tissue, [7,36], as well as implementing numerous dose reduction strategies in pediatric interventional cardiology [3,37]. The difficulties associated with cancer and non-cancer risk assessments from pediatric radiation exposure could be partly overcome with precise dose estimation and biochemical studies to better understand the mechanisms that underly the development of disease processes and provide indicators of risk [16,17,18]. 

Radiation can induce DNA damage, especially DNA double-strand breaks (DSBs), which are the most lethal type of DNA damage and can result in mutations, chromosomal abnormalities, and the further development of cancer, as well as other severe health effects. However, the full spectra of biological mechanisms underlying the adverse health effects after irradiation are only partly understood. It has been reported that several biological pathways, such as DNA repair, inflammatory response, oxidative stress induction, as well as metabolic changes, are involved in response to IR exposure [17,38]. A multifactorial approach in conjunction with robust high-throughput technologies and integrated computational approaches, such as systems biology, may facilitate the discovery of new pathways and biomarkers for the prediction of long-term adverse health effects of IR exposure [17,38,39]. 

Accordingly, biological research on the HARMONIC project will investigate the changes induced by medical radiation at the level of specific biomarkers which might be considered ‘early signs’ of tissue damage before the full development of adverse health effects. The project will focus on changes related to oxidative stress [19,20], inflammation [22,23], as well as nuclear and mitochondrial DNA damage [25,26], in order to identify the long-term health risks [21,24,27,28,29].

In parallel, the study will also use high-resolution approaches of proteomics and whole miRNA transcriptomes to provide an integrated assessment of bio-molecular responses to pediatric radiation exposure by identifying biological pathways that may underlie the adverse health effects of the exposures.

Of note, this study has a longitudinal design which will allow the shorter-term and longer-term biological response of IR to be compared. To account for the differences between the participants, we will anchor the data of each individual on their own baseline data. This will allow us to measure the treatment effects on an individual level immediately and up to 1 year later. 

An additional aspect of this study is the comparison of biomarkers in saliva with those in blood samples. Especially in vulnerable populations, such as children [40,41], saliva offers an attractive non-invasive sampling method that is relatively inexpensive, safe, and easy to use. Saliva could be a valuable alternative as a biological source for human biomonitoring in occupational and environmental medicine [41], but further studies are needed to explore the robustness, reproducibility, and validity of salivary biomarkers in comparison to those analyzed in blood.

As the study includes a cohort of pediatric patients that will be well characterized in terms of dosimetry to “in-field” and “out-of-field” organs [33,34,35] (https://harmonicproject.eu, accessed on 4 March 2023), it will be possible to investigate dose–response relationships between biomarkers and doses to organs for both radiotherapy and interventional cardiology, as well as a comparison of effects of radiation quality. 

The HARMONIC databases will register the individual responses for a defined set of biomarkers and facilitate studies on the mechanisms that underly radiation-induced second/primary cancers, as well as cardiac and vascular damages. A mechanistic understanding, together with biomarkers for individuals at increased risk, has the potential to increase the power of epidemiological studies regarding the health effects of different radiotherapy modalities. 

Hence, we believe that a better understanding of the underlying biological and cellular mechanisms will complement the epidemiological approach of the HARMONIC project (https://harmonicproject.eu/, accessed on 4 March 2023). This will provide a unique opportunity to gain better insight into the biological effects of medical radiation doses in pediatric patients. 

In summary, the findings of this research project hold the potential to provide mechanistic insight in the molecular and cellular responses involved in the effects of IR of different radiation quality and doses. The HARMONIC project aims to improve the protection of patients and maximize the benefits from medical applications. The final expected output from the biological part of the project will be able to define the predictive biomarkers to be used for molecular epidemiology studies in order to identify patients at higher risk for adverse health effects.

## Figures and Tables

**Table 1:** Inclusion and exclusion criteria.

Radiotherapy	Interventional Cardiology
**Inclusion criteria**	
▪ Age at diagnosis ≤ 21 years ▪ Informed consent of parent/guardian as well as child/patient ▪ Patients treated for: brain tumors (except malignant gliomas); head and neck tumors (e.g., rhabdomyosarcomas and nasopharyngeal carcinoma); Hodgkin’s lymphoma ▪ Patients receiving pulmonary and chest radiation for: Ewing sarcoma; other chest sarcomas; lung metastasis of Wilms and Ewing tumors; other tumors ▪ Patients receiving craniospinal radiation therapy for: Medulloblastoma or other tumors	▪ Age of patients: 5–22 years ▪ Patients with congenital heart disease ▪ Informed consent of parent/guardian as well as child/patient
**Exclusion criteria**	
▪ Chromosomal abnormalities and/or genetic syndromes ▪ Absence of informed consent	

## Data Availability

No new data were created or analyzed in this study. Data sharing is not applicable to this article.

## Abbreviations

mtDNAcn	mtDNA copy number
8-Oxo-dG	8-hydroxy-2′-deoxyguanosine
IL-6	interleukin-6
IL-10	interleukin-10
TNF-α	tumor necrosis factor-α
MCP-1	monocyte chemoattractant protein-1
NF-kB	nuclear factor kappaB
PBMC	peripheral blood mononuclear cell
PTX3	pentraxin 3
PBMC	peripheral blood mononuclear cells
miRNA	microRNA
Lym	cell pellet/lymphocytes
PCP	plasma from CPT
P	plasma from EDTA
B	rest of blood
S	serum
SC	blood clot
Sal	saliva
